# Early-infantile onset epilepsy and developmental delay caused by bi-allelic *GAD1* variants

**DOI:** 10.1093/brain/awaa178

**Published:** 2020-07-23

**Authors:** Caroline Neuray, Reza Maroofian, Marcello Scala, Tipu Sultan, Gurpur S Pai, Majid Mojarrad, Heba El Khashab, Leigh deHoll, Wyatt Yue, Hessa S Alsaif, Maria N Zanetti, Oscar Bello, Richard Person, Atieh Eslahi, Zaynab Khazaei, Masoumeh H Feizabadi, Stephanie Efthymiou, SYNaPS Study Group, Stanislav Groppa, Blagovesta Marinova Karashova, Wolfgang Nachbauer, Sylvia Boesch, Larissa Arning, Dagmar Timmann, Bru Cormand, Belen Pérez-Dueñas, Gabriella Di Rosa, Jatinder S Goraya, Tipu Sultan, Jun Mine, Daniela Avdjieva, Hadil Kathom, Radka Tincheva, Selina Banu, Mercedes Pineda-Marfa, Pierangelo Veggiotti, Michel D Ferrari, Alberto Verrotti, Giangluigi Marseglia, Salvatore Savasta, Mayte García-Silva, Alfons Macaya Ruiz, Barbara Garavaglia, Eugenia Borgione, Simona Portaro, Benigno Monteagudo Sanchez, Richard Boles, Savvas Papacostas, Michail Vikelis, Eleni Zamba Papanicolaou, Efthymios Dardiotis, Shazia Maqbool, Shahnaz Ibrahim, Salman Kirmani, Nuzhat Noureen Rana, Osama Atawneh, George Koutsis, Marianthi Breza, Salvatore Mangano, Carmela Scuderi, Eugenia Borgione, Giovanna Morello, Tanya Stojkovic, Massimi Zollo, Gali Heimer, Yves A Dauvilliers, Pasquale Striano, Issam Al-Khawaja, Fuad Al-Mutairi, Hamed Sherifa, Hala T El-Bassyouni, Doaa R Soliman, Selahattin Tekes, Leyla Ozer, Volkan Baltaci, Suliman Khan, Christian Beetz, Khalda S Amr, Vincenzo Salpietro, Yalda Jamshidi, Fowzan S Alkuraya, Henry Houlden

**Affiliations:** a1 UCL Queen Square Institute of Neurology, University College London, London, UK; a2 Department of Neurology, Christian Doppler Klinik, Paracelsus Medical University, Salzburg, Austria; a3 Department of Neurosciences, Rehabilitation, Ophthalmology, Genetics, Maternal and Child Health, University of Genoa, Genoa, Italy; a4 IRCCS Istituto Giannina Gaslini, Genoa, Italy; a5 Department of Pediatric Neurology, Children’s Hospital and Institute of Child Health, Lahore, Pakistan; a6 Medical University of South Carolina, USA; a7 Department of Medical Genetics, Faculty of Medicine, Mashhad University of Medical Sciences, Mashhad, Iran; a8 Medical Genetics Research Center, Mashhad University of Medical Sciences, Mashhad, Iran; a9 Genetic Center of Khorasan Razavi, Mashhad, Iran; a10 Department of Pediatrics, Children’s Hospital, Ain Shams University, Cairo, Egypt; a11 Department of Pediatrics, Dr. Suliman Al Habib Medical Group, Riyadh, Saudi Arabia; a12 Structural Genomics Consortium, Nuffield Department of Clinical Medicine, University of Oxford, UK; a13 Student Research Committee, Faculty of Medicine, Mashhad University of Medical Sciences, Mashhad, Iran; a14 Department of Clinical and Experimental Epilepsy, University College London, London, UK; a15 GeneDx, Gaithersburg, MD, USA; a16 Clinical Genetics Department, National Research Centre, Cairo, Egypt; a17 Department of Pediatrics, Faculty of Medicine, Benha University, Benha, Egypt; a18 Dicle University, School of Medicine, Department of Medical Genetics, Diyarbakir, Turkey; a19 Yuksek Ihtisas University, School of Medicine, Department of Medical Genetics, Ankara, Turkey; a20 Mikrogen Genetic Diagnosis Center, Ankara, Turkey; a21 CENTOGENE AG, Rostock; a22 Molecular Genetics Department, National Research Centre, Cairo, Egypt; a23 Molecular and Clinical Sciences Institute St George’s, University of London, UK; a24 Department of Genetics, King Faisal Specialist Hospital and Research Center Riyadh, Saudi Arabia

**Keywords:** GAD1, epilepsy, neurodevelopmental delay, muscle weakness, cleft palate

## Abstract

Gamma-aminobutyric acid (GABA) and glutamate are the most abundant amino acid neurotransmitters in the brain. GABA, an inhibitory neurotransmitter, is synthesized by glutamic acid decarboxylase (GAD). Its predominant isoform GAD67, contributes up to ∼90% of base-level GABA in the CNS, and is encoded by the *GAD1* gene. Disruption of *GAD1* results in an imbalance of inhibitory and excitatory neurotransmitters, and as *Gad1*^−/−^ mice die neonatally of severe cleft palate, it has not been possible to determine any potential neurological dysfunction. Furthermore, little is known about the consequence of GAD1 disruption in humans. Here we present six affected individuals from six unrelated families, carrying bi-allelic *GAD1* variants, presenting with developmental and epileptic encephalopathy, characterized by early-infantile onset epilepsy and hypotonia with additional variable non-CNS manifestations such as skeletal abnormalities, dysmorphic features and cleft palate. Our findings highlight an important role for *GAD1* in seizure induction, neuronal and extraneuronal development, and introduce *GAD1* as a new gene associated with developmental and epileptic encephalopathy.

## Teaser

Mice lacking *GAD1* show neonatal mortality, but the human phenotype associated with *GAD1* disruption is poorly characterized. Neuray *et al.* describe six patients with biallelic *GAD1* mutations, presenting with early-infantile onset epilepsy, neurodevelopmental delay, muscle weakness and non-CNS manifestations.

## Introduction

The neurotransmitter γ-aminobutyric acid (GABA) is one of the main inhibitory neurotransmitters deriving from glutamate (Cooper *et al.*, 1996). It plays a critical signalling role in the nervous system and also in a number of non-neuronal cell types. The enzyme responsible for the conversion of glutamate into GABA is glutamate decarboxylase (GAD), and occurs in two isoforms GAD65 and GAD67, depending on its molecular weight (Kaufman *et al.*, 1991). These isoforms are products of two different genes, *GAD1* (encoding a 67 kDa molecular weight protein, GAD67) and *GAD2* (encoding a 65 kDa molecular weight protein, GAD65). GAD67 is constitutively active and produces >90% of the base level GABA in the CNS, whilst GAD65 is transiently activated (Asada *et al.*, 1997).

Animal studies have also shown a distinct role for GAD65 and GAD67. *Gd65*^−/−^ mice are viable but show a higher susceptibility to seizure induction despite normal GABA levels (Asada *et al.*, 1996), whereas *Gad67*^−/−^ mice are characterized by neonatal death, severe cleft plate and respiratory failure. GAD activity and GABA concentration are also drastically reduced in *Gad67*^−/−^ mice (Asada *et al.*, 1997).

The severity of the *Gad67*^−/−^ phenotype in animal models might suggest severe phenotypical manifestions in humans, yet there have been few reported families with *GAD1* mutations (Lynex *et al.*, 2004; Saito *et al.*, 2010; Curley *et al.*, 2011; Ruzicka *et al.*, 2015; Magri *et al.*, 2018). Previous reports have described seemingly unparalleled phenotypes, which include schizophrenia, autism spectrum disease and cerebral palsy, and functional studies are missing to confirm the pathogenicity of these reported mutations.

Here we report a series of six affected individuals with distinct phenotypical features from six unrelated families with bi-allelic mutations in the *GAD1* gene (three carrying homozygous missense mutations, one carrying a homozygous frameshift variant, two compound heterozygous variants, and one harbouring a homozygus stop gain variant). All affected individuals presented with seizures, strongly impaired neurocognitive development, and reduced muscle tone of variable severity. Interestingly only one presented with cleft palate, which has been suggested to be one of the key features in the *GAD1* animal models.

## Material and methods

### Patients and genetic analysis

Six patients from six unrelated families of Persian (Family A), Pakistani (Family B), African American (Family C), Sudanese (Family D), Egyptian (Family E) and Turkish ancestry (Family F) were identified through GeneMatcher (Sobreira *et al.*, 2015) and enrolled in this study. The study was conducted according to the Declaration of Helsinki and with the approval of the institutional review boards of University College of London and participating centres. Genetic testing through whole exome sequencing (WES) was carried out in different research centres after informed consent was obtained from the parents or legal guardians of the studied subjects. Genomic DNA was extracted from peripheral blood obtained from the probands, parents, and unaffected siblings (when available). Exome sequencing and data analysis was performed as follows: Families A, B and D in the according centres as previously described (Monies *et al.*, 2019; Dias *et al.*, 2019), Family C through GeneDx (Retterer *et al.*, 2016), and Families E and F at Centogene (Bauer *et al.*, 2018). Potential candidate causal variants were subsequently confirmed by independent bi-directional Sanger sequencing. Detailed information is provided in the Supplementary material.

### Data availability

The data that support the findings of this report are available from the corresponding author, upon reasonable request.

## Results

### Clinical manifestation

Four of the affected individuals were born from consanguineous parents (first or second cousins), and all were born at term following a normal pregnancy. A key clinical feature common to all affected individuals was early onset seizures (from 2 to 6 months), predominantly focal motor seizures with and without impaired awareness (two with additional epileptic spasms, four with focal non-motor seizures, and five with bilateral motor seizures). Seizures were pharmacologically controlled in three of six affected individuals, with three reported as drug-resistant. Drug regimens differed across the individuals. EEG at seizure onset showed a burst suppression pattern in two individuals, diffuse slowing with multifocal as well as generalized sharp waves in two, and hypsarrhythmia in two. Follow-up EEGs showed diffuse slowing of background activity or persistent epileptic activity (two of six). Cranial MRI was normal in all but two individuals, one showing slight ventricular enlargement and one moderate global atrophy.

The second common clinical feature was severe developmental delay. Most patients did not achieve any speech or non-verbal communication, only one was reported to have developed simple speech and basic perceptive language skills. This individual has remained seizure-free under medication. Of the more severely affected individuals, despite remaining seizure-free following pharmacological intervention, they still failed to accelerate in their intellectual development.

The third key feature we observed was a reduced muscle strength (five of six individuals) of varying severity ranging from slight muscle tone (one of five) to limited head control, inability to sit or crawl (four of six) and nasogastric tube dependence, due to dysphagia (two of six). While slight dysmorphic facial features were seen in four of six individuals (Table 1), only one presented with a cleft palate.

**Table 1:** Clinical features of *GAD1* patients

Family	A (Patient III-2)	B (Patient III-4)	C (Patient II-1)	D (Patient II-4)	E (Patient III-3)	F (Patient II-2)
Sex/ ethnic origin	Female/ Persian	Male/ Pakistani	Male/ African American	Male/ Sudanese	Female/ Egyptian	Female/ Turkish
Consanguinity	Yes (double first cousins)	Yes	–	Yes (second cousins)	Yes (first cousins)	Yes (first cousins)
Age at first/last exam	5 y/10 y 3 m	6 m/7 y	2 m/22 m	2 m/18 m	6 m/4 y	1 m/3 y
**Development**						
Milestones	Delayed in all milestones, simple speech at 4 y, walking delayed, no complex movements	Delayed in all milestones, sitting and crawling	Delayed in all milestones, no head control, no sitting, no speech	Severe delay, poor head control achieved at 18m, no sitting	Severe delay in all milestones, bed ridden	Severe delay in all milestones, no sitting or crawling
ID	Moderate	Severe	Severe	Severe	Severe	Severe
Vision/hearing	High myopia	Normal	NA	Normal	Moderate hearing impairment	High myopia
Dysmorphic facial features	Yes	Yes	–	Yes	Yes	–
Cleft palate	–	–	–	–	Yes (surgical correction)	–
Skeletal abnormalities	Clindodactyly, pes planus, scoliosis	Arthrogryposis of lower limbs	–	Short arms	Congenital hip dislocation and malformation	
Neurological examination	Mild hypotonia	Brisk DTR, stereotypic hand movements, oral automatisms	Mild hypotonia, spasticity in lower extremities, oropharyngeal dysphagia	Severe hypotonia, dysphagia (floppy epiglottis)	Severe hypotonia, hyporeflexia, dysphagia	Severe hypotonia
**Epilepsy**						
Age at onset	2 m	6 m	2 m	2 m	6 m	2 m
Type(s) of seizure at onset	Focal non/motor with impaired awareness	Focal motor impaired awareness, bilateral tonic-clonic	Focal non/motor with impaired awareness, bilateral tonic clonic	Epileptic spasms	Bilateral tonic clonic	Focal ± to bilateral motor with impaired awareness
Seizure progression (age)	Controlled (10 y), last seizure at age 7 y	Controlled (7 y), last seizure at 5.5 y	Refractory (28 m)	Spasms continue, seizures controlled (18 m)	Refractory (4 y)	Partial control, 1 seizure/week
EEG at onset	Burst suppression	Multifocal and generalized epileptogenic activity	Burst suppression	Hypsarrhythmia	Generalized epileptogenic activity	Hypsarrhythmia
Follow-up EEG (age)	Normal (11 y)	Normal (7 y)	Slowing, multifocal epileptic discharges (28 m)	No epileptic abnormalities (18 m)	NA	Generalized epileptiform activity (4 m)
AEDs trialled	PB, *VPA*, *CBZ*, *CLB*	PB, VPA, *CLZ*	*CBD*,* vigabatrin*,* ketogenic diet*,* CLZ*, PB	steroids, TP, *vigabatrin*	*LEV*, *CLZ*, TP	*PB*, *TP*, *LEV*, CLZ, vigabatrin, steroids
Cardiovascular MRI (age)	Normal (5 y)	Prominent ventricular space (6 m)	Normal (2 m)	Normal (6 m)	Moderate global atrophy (1 y)	Normal (2 m)
**Other features**	Hydronephrosis, nephrocalcinosis, bilateral kidney stones	–	NG-tube dependent	Diastasis recti	Intermittent NG tube dependence	–

Current antiepileptic drugs (AEDs) are highlighted in italics. CBD = cannabidiol; CBZ = carbamazepine; CLB = clobazam; CLZ = clonazepam; DTR = deep tendon reflexes; ID = intellectual disability; LEV = levetiracetam; NA = not applicable; NG = nasogastric; OXC = oxcarbazepine; PB = phenobarbital; TP = topiramate; VPA = valproic acid.

**Figure 1:**
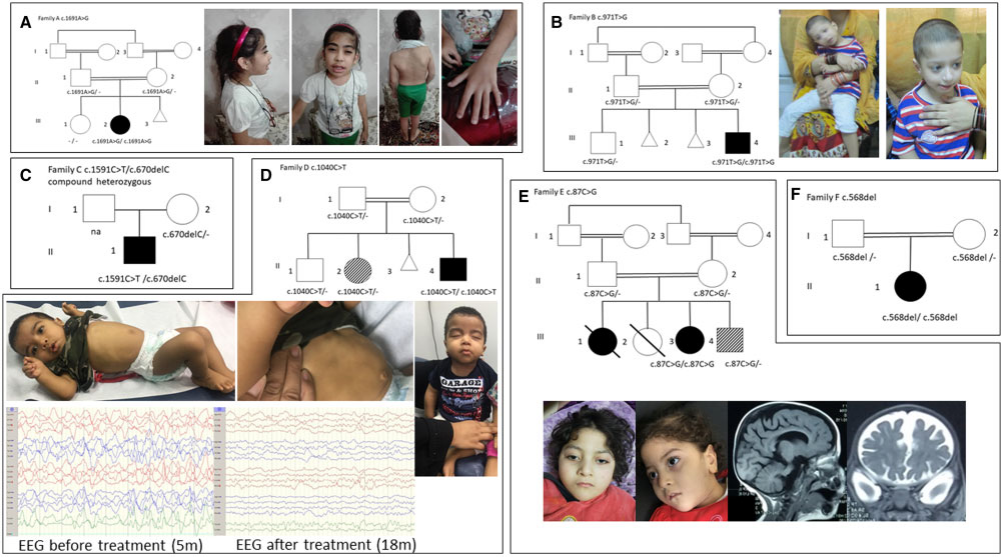
**Pedigrees of the reported families and clinical pictures of *GAD1* patients.** (**A**) The female patient from Family A (Patient II-2) carries the homozygous c.1691A>G p.(Asn564Ser) variant and shows dysmorphic features with thick eyebrows, protruding ears, scoliosis, and long fingers with clinodactyly. (**B**) Patient from Family B (Patient III-4) harbours the c.971T>G p.(Phe324Cys) variant. He has slight dysmorphic features (wide mouth, thin upper lips, bitemporal narrowing and retrognathia). (**C**) Pedigree showing the segregation of the compound heterozygous variants c.1591C>T, c.1591C>T p.(Arg531*) and c.670delC p.(Leu224Serfs*5) in Family C. (**D**) Pedigree of Family D shows the segregation of the c.1040C>T p.(Thr347Met) variant. Patient II-4 carries the variant in homozygous state. He is severely hypotonic and shows severe dysmorphic features (infra-orbital creases, severely depressed nasal bridge, anteverted nares, prominent nasolabial folds). In addition, significant diastasis recti can be observed. His sister (Patient II-2) is heterozygous for the same variant and suffers from a different neurodevelopmental condition without seizures. (**E**) Patient III-2 from Family E harbours the c.87C>G (Tyr29*) variant, severely affected with dysmorphic facial features and global atrophy on cardiovascular MRI, one similarly affected sibling passed away without any genetic testing being performed, another sibling passed away only a few hours after birth, no phenotypical or genetic assessment could be carried out, and one sibling is alive with a different phenotype (sensoneural hearing loss, Hirschsprung disease). (**F**) Patient II-1 from Family F harbours the c.568del (Gln190Serfs*11) variant. His parents are both heterozygous carriers of the same variant. Empty and full symbols represent healthy and affected individuals, respectively. The symbol with diagonal lines indicates carrier status/different phenotype. The double line indicates consanguinity.

Other features that were observed without clear common elements included hirsutism, kidney stones, urogenital malformations, diastasis recti, reduced head circumference, clinodactyly, short arms, arthrogryposis of the lower limbs and congenital hip dislocation*.* Metabolic workups did not show any abnormalities. A detailed clinical summary of all affected individuals can be found in Table 1 along with images of key features and family pedigrees in Fig. 1.

### Genetic findings

Variants were prioritized in each family based on allele frequency <0.01%, predicted impact on protein function, and biological consistency. Potentially causal bi-allelic variants in *GAD1* were identified in all affected individuals. The segregation of the variants with the clinical phenotype was confirmed by Sanger sequencing, which showed a recessive mode of inheritance. Detailed genetic results are provided in Table 2. All affected individuals carried ultrarare *GAD1* variants, which were predicted to result in impaired protein function. Homozygous variants were identified in five families (Families A, B, D, E and F), whereas compound heterozygous variants were found in Family C (Table 2).

**Table 2:** Frequency and predicted effect of the reported *GAD1* variants

GAD1 variant [NM_000817.2]	c.87C>G (p.Tyr29Ter) (Family E)	c.568delC (p.Gln190Serfs Ter11) (Family F)	c.670delC p.(Leu224Serfs*5) (Family C)	c.971T>G p.(Phe324Cys) (Family B)	c.1040C>T p.(Thr347Met) (Family D)	c.1591C>T p.(Arg531*) (Family C)	c.1691A>G p.(Asn564Ser) (Family A)
g. (hg19)	g.171678601C>G	g.171693323delC	g.171700586delC	g.171702542T>G	g.171704223C>T	g.171715383C>T	g.171716298A>G
Internal database	–	–	–	–	–	–	–
ExAC/GnomAD	–	–	–	0.00000796 (2 het)	0.00000795 (2 het)	0.00000398 (1 het)	0.00000398 (1 het)
GME	–	–	–	–	–	–	–
Iranome	–	–	–	–	–	–	–
Ensembl	–	–	–	–	–	–	–
SIFT	D- N/A	N/A	N/A	D (0.9125)	D (0.9125)	N/A	D (0.9125)
MutationTaster	DC (1)	DC (1)	DC (1)	DC (0.9768)	DC (1)	DC (1)	DC (1)
PolyPhen-2	N/A	N/A	N/A	PD (0.978)	PD (1)	N/A	PD (1)
GERP score	4.97	5.55	6.17	5.91	5.67	4.67	5.48
CADD score	35	N/A	N/A	28	29.1	43	26.1
ACMG class	5 (PVS1, PM2, PP4)	5 (PVS1, PM2, PP3)	5 (PVS1, PM2, PP3)	3 (PM2, PP3)	3 (PM2, PP3)	5 (PVS1, PM2, PP3)	3 (PM2, PP3)
GeneDx	0	0	0/135 084	0	1/130 874	0/135 084	1/130 874

CADD = Combined Annotation Dependent Depletion; D = damaging; DC = disease causing; GeneDx = variant frequencies from the GeneDx database; GERP = Genomic Evolutionary Rate Profiling; GnomAD = Genome Aggregation Database; GME = Greater Middle East (GME) Variome Project; het = heterozygous; N/A = not applicable; PD = probably damaging; PM2 = Pathogenic Moderate 2; PP3 = Pathogenic Supporting 3; PVS1 = Pathogenic Very Strong 1; SIFT = Sorting Intolerant From Tolerant.

**Figure 2:**
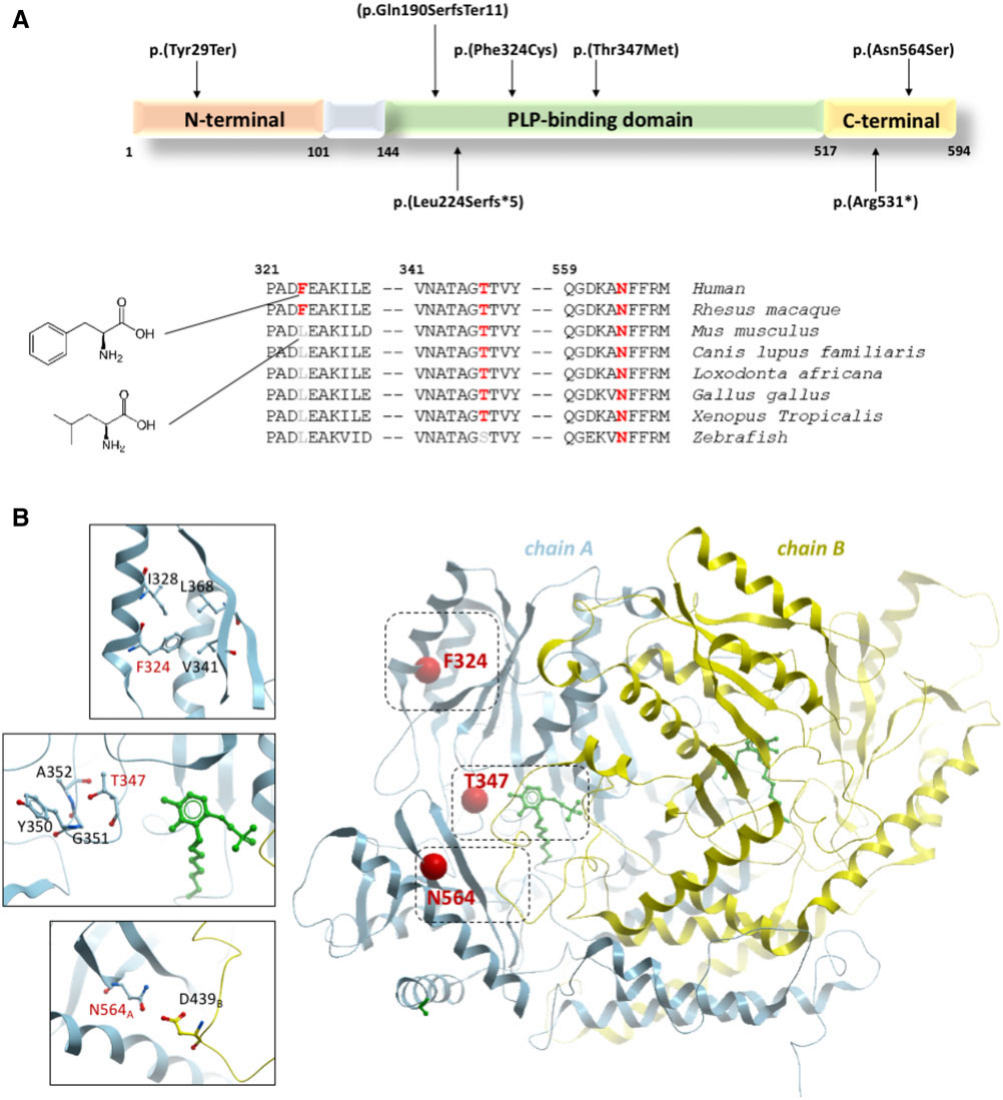
**Schematic and cartoon representation of GAD1.** (**A**) Schematic representation of the GAD1 isoform GAD67 (NP_000808.2) with the pathogenic variants identified in this study. Of the six variants, four fall within the PLP-binding domain, a conserved region that is essential for the binding of the crucial cofactor pyridoxal 5′-phosphate (PLP). The remaining variants affect the C-terminal domain, which contains the catalytic site of the enzyme. Conservation status among different species is shown for the missense variants. (**B**) Cartoon representation of human GAD1 dimer (PDB: 2okj) with the two subunits in blue and yellow. Sites of the three missense mutations in this study are shown as red spheres, and close-up views of their nearby atomic environment are shown as *insets*. The PLP cofactor is shown in green.

The affected proband from Family A carried a homozygous c.1691A>G, p.(Asn564Ser) variant, which is reported only once in the heterozygous state in gnomAD. This variant has a combined annotation dependent depletion (CADD) score of 26.1 and is predicted to be pathogenic by several bioinformatic prediction tools, including SIFT (score 0.9125), MutationTaster, and PolyPhen-2 (score 1) (Table 2). The proband from Family B harboured a homozygous c.971T>G, p.(Phe324Cys) variant, which causes the substitution of a phenylalanine residue at position 324 with cysteine. This position is not strictly conserved in other species, where leucine is found in place of phenylalanine. However, both phenylalanine and leucine belong to the class of the amino acids with long hydrophobic chains (Fig. 2A) and share several chemical features. This variant has been seen in the heterozygous state in gnomAD with a minor allele frequency of 0.00000796. It has a CADD score of 28 and is predicted damaging by all the prediction tools used (scores of 0.9125 and 0.978 for SIFT and PolyPhen-2, respectively). The proband from Family D carried a homozygous c.1040C>T, p.(Thr347Met) variant, which was reported twice in the heterozygous state in the gnomAD database. It was predicted damaging by both SIFT and PolyPhen-2 with high scores (0.9125 and 1, respectively). The CADD score for this variant was 29.1. Further *in silico* analysis predicted a reduction in protein stability for p.(Phe324Cys) and p.(Asn564Ser), in association with a break in H-bonds in the pyridoxal 5′-phosphate (PLP) binding domain for p.(Thr347Met). Both these changes might result in an impairment of the protein function due to the abnormal degradation or the decreased binding activity towards PLP, leading to a likely loss-of-function effect (Fig. 2B and Supplementary Table 1). In Family E, a homozygous stop gain variant c.87C>G, p.(Tyr29Ter) was identified. This variant is absent in all the queried population datasets. It is predicted damaging by SIFT (score not available) and disease-causing by Mutation Taster, with a CADD score of 35. This null variant likely causes a nonsense-mediated mRNA decay (NMD), leading to a complete loss-of-function. The proband from Family F carries a homozygous c.568delC p.(Gln190SerfsTer11) variant, which is absent in gnomAD and other population datasets queried. It is predicted damaging by MutationTaster (score 1) and classified as pathogenic according to the ACMG guidelines. Family C was the only family in which heterozygous variants were found: c.1591C>T, p.(Arg531*) and c.670delC, p.(Leu224Serfs*5). This individual’s mother was a carrier for c.670delC; however, the individual’s father was not available for genetic testing. The c.1591C>T, p.(Arg531*) variant results in a stop gain with a very high likely impact on protein function, as highlighted by a CADD score of 43. This is only reported in heterozygous state in the gnomAD database. Similarly, the frameshift variant c.670delC, p.(Leu224Serfs*5) is predicted to be disease-causing by MutationTaster and affects a very conserved residue, with a GERP score of 6.17. The null variants identified in Families B, E, and F may cause a premature termination of the transcript leading to a truncated protein or, alternatively, affected transcripts might be target of NMD.

## Discussion

In humans, *GAD1* encodes a 67-kDa protein, GAD67, which is the major contributor to GABA production in the CNS. As the major embryonic GAD isoform, it also plays a pivotal role in synaptogenesis and neuronal development (Asada *et al.*, 1997; Soghomonian and Martin, 1998; Sgadò *et al.*, 2011).

The 594 amino acid protein GAD67 is composed of a N-terminal domain involved in the generation of GAD65–GAD67 heterodimers and subcellular targeting, a C-terminal domain containing the catalytic site, and a central conserved domain binding PLP (Bosma *et al.*, 1999; Martin *et al.*, 2000; Lynex *et al.*, 2004). This pyridoxal-dependent decarboxylase domain is essential for GAD67 function as GAD requires PLP as a cofactor to catalyze the generation of GABA from glutamate (Lernmark, 1996). Four of the seven variants identified in our families affect conserved residues in the PLP-binding domain, likely leading to loss-of-function (Fig. 1A). In particular, the two missense variants c.971T>G, p.(Phe324Cys) and c.1040C>T, p.(Thr347Met) probably cause impaired PLP binding, whereas the null variants c.568delC, p.(Gln190SerfsTer11) and c.670delC p.(Leu224Serfs*5) might result in a truncated protein or NMD. Similarly, the c.87C>G, p.(Tyr29Ter) variant localized to the N-terminal domain results in a premature stop codon, likely leading to NMD. The remaining two variants, c.1591C>T, p.(Arg531*) and c.1691A>G, p.(Asn564Ser), are localized to the C-terminal domain of the protein and are predicted to result in a complete and partial loss-of-function of the catalytic activity, respectively (Fig. 2B). According to gnomAD, *GAD1* shows a moderate intolerance to missense variants (*Z*-score = 2.32; observed 217 and expected 336.5) and predicted loss-of-function variants (expected 33.1, observed 9). The likely negative impact of the missense variants on PLP-binding and C-terminal catalytic domains, together with the finding of four null pathogenic variants in our case series, supports the idea that the clinical phenotypes observed are likely due to a loss-of-function mechanism.

With regard to the possible genotype-phenotype correlation, there have been few clinical case reports of *GAD1* mutations, with little overlap in clinical features (Lynex *et al.*, 2004; Ruzicka *et al.*, 2015; Magri *et al.*, 2018). The six patients reported here, however, do show a clear phenotypical manifestation.

One of the main common features we observed was the occurrence of seizures at a young age (between 2 and 6 months of age). Since GAD1 is centrally involved in the production of GABA, *GAD1* mutations likely lead to an imbalance of GABA in the brain. Previous studies have shown that abnormalities in GABAergic function play an important role in seizure induction (Olsen and Avoli, 1997). Dysfunction of the GABAergic system can be caused through either abnormal GABA synthesis (e.g. GAD dysfunction) or abnormal signalling (e.g. GABA receptor malfunction). Animal studies have shown that mutant mice lacking GAD or certain subunits of GABA-A receptors are prone to spontaneous epileptic seizures (Asada *et al.*, 1996; Kash *et al.*, 1997; DeLorey *et al.*, 1998). It has also been shown that there is a reduction of GABAergic neurons in the epileptic brain, independent of the seizure’s aetiology supporting a conclusion that seizures themselves further decrease GABA release within the brain, causing further imbalance (Wang *et al.*, 2011). This evidence suggests that there is a clear correlation between abnormalities within the GABAergic system and seizure occurrence. The occurrence of seizures early in life of our patients is therefore not a surprising phenotypical finding. However, previous data suggest that the intellectual development of newborns with epileptic encephalopathies is strongly dependent on seizure control, and seizure freedom usually leads to acceleration of intellectual development (Bombardieri *et al.*, 2010). However, despite seizure control (seizure freedom achieved in three of six individuals), all patients described here still showed severe intellectual disability. This has been observed previously in other genetic conditions (Weckhuysen *et al.*, 2012; Berecki *et al.*, 2019), leading to the assumption that genetic defects themselves are influencing development and cognition, independently from seizure control. This is reflected in the ILAE’s terminology of ‘developmental and epileptic encephalopathies’, though a clear distinction between epileptic and developmental encephalopathy has been advised (Scheffer *et al.*, 2017; Scheffer and Liao, 2020). Furthermore, some EEG abnormalities continued to be prominent after achieving seizure freedom. We therefore hypothesize that *GAD1* mutations may also play an important role in intellectual development.

The reduced muscle tone and weakness, causing severe disability in four of six affected individuals was a surprising finding. There is a single case described in the literature of an individual with cerebral palsy and a *GAD1* variant (Lynex *et al.*, 2004). However, identification of the variant was based on autozygosity mapping, in which they identified a recessive locus of 5 cM located at 2q24-31.1. The investigators subsequently investigated the most interesting candidate in that region by sequencing the exons of *GAD1* and identified a homozygous missense variant c.35C>G (p.Ser12Cys) (NM_013445.3) in the N-terminal domain of the protein (Lynex *et al.*, 2004). This variant is rare in gnomAD (exomes allele frequency of 0.0000239) and absent in the homozygous state. However, the predictions on its pathogenicity are conflicting as it is predicted benign by MutationAssessor, DEOGEN2, and MetaLR. Furthermore, the 2q24-31.1 locus encompasses several other possible genes of interest (e.g. *DYNC1I2*, responsible for a neurodevelopmental disorder characterized by intellectual disability, spasticity, and neuroradiological anomalies), which were not investigated. The N-terminal domain of GAD67 is involved in the generation of GAD65–GAD67 heterodimers and subcellular targeting, and whilst we cannot rule out a possible impairment of the interaction of GAD67 with GAD65 or an alteration in GAD67 subcellular targeting as a result of this variant, we suggest that variants affecting the PLP-binding and C-terminal domain cause a more severe deficiency in GAD67 activity.

In addition, we also identified four null variants likely resulting in a loss-of-function. According to these observations and the consistency of the phenotype in our case series, we emphasize that *GAD1* pathogenic variants should be considered the cause of a distinctive neurodevelopmental disorder instead of spastic cerebral palsy (Lynex *et al.*, 2004). The clinical observation of muscle weakness is particularly interesting as it has not been described previously. Other GAD1-related diseases, such as antibody-mediated syndromes, have been associated with motor symptoms (Dayalu and Teener, 2012). However, these motor phenomena are usually linked to a hyperexcitability (increased muscle tone leading to rigidity, muscle spasms, and stiff person syndrome), as well as other neurological symptoms (e.g. Miller Fisher syndrome, eye movement disorders, cerebellar ataxia, epilepsy, limbic encephalitis, etc.) (Moersch and Woltman, 1956; Dalakas *et al.*, 2000; Saiz *et al.*, 2008; Tohid, 2016). Of note, in no case has muscle weakness been linked to GAD1 deficiency.

While *Gad67*^−/−^ mice have been reported to die within the first hours of life due to a cleft palate (Asada *et al.*, 1997; Condie *et al.*, 1997), only one of our patients (Family E, Patient III-3) was born with a cleft palate (the same patient also showed congenital bilateral hip dislocation with shallow acetabulum, talipes equinovarus and hearing impairment.) Several studies have hypothesized that the development of a cleft palate is linked to reduced tongue movement during embryonic development and therefore secondary to CNS dysfunction (Iseki *et al.*, 2007; Oh *et al.*, 2010; Saito *et al.*, 2010). *Gad1* expression has also been shown in different non-neural tissues, such as the tail bud, limb mesenchyme, vibrissal placodes, and pharyngeal arches in various stages of embryonic development (Maddox and Condie, 2001). This observation has suggested a broader influence of GAD1 and GABA function on non-neural development (Maddox and Condie, 2001), supporting a possible primary role of GAD1 impaired function in the pathogenesis of non-neural defects. However, further studies are required to confirm this in humans.

In conclusion, this case series reports distinct phenotypical features caused by *GAD1* variants, including early-infantile onset epilepsy, severe developmental delay and muscle weakness. Less consistent features include skeletal abnormalities and dysmorphic facial features, including cleft palate. Functional studies and larger clinical series will be necessary to further assess genotype-phenotype correlations for *GAD1* variants.

## Supplementary Material

awaa178_Supplementary_Data

## Appendix 1

Consortia and network members involved in this study are listed below. The full details are available in the Supplementary material.

### The Synaptopathies and Paroxysmal Syndromes (SYNaPS) Study Group

http://neurogenetics.co.uk/synaptopathies-synaps/

Stanislav Groppa, Blagovesta Marinova Karashova, Wolfgang Nachbauer, Sylvia Boesch, Larissa Arning, Dagmar Timmann, Bru Cormand, Belen Pérez-Dueñas, Gabriella Di Rosa, Jatinder S. Goraya, Tipu Sultan, Jun Mine, Daniela Avdjieva, Hadil Kathom, Dr Radka Tincheva, Selina Banu, Mercedes Pineda-Marfa, Pierangelo Veggiotti, Michel D. Ferrari, Alberto Verrotti, Giangluigi Marseglia, Salvatore Savasta, Mayte García-Silva, Alfons Macaya Ruiz, Barbara Garavaglia, Eugenia Borgione, Simona Portaro, Benigno Monteagudo Sanchez, Richard Boles, Savvas Papacostas, Michail Vikelis, Eleni Zamba Papanicolaou, Efthymios Dardiotis, Shazia Maqbool, Shahnaz Ibrahim, Salman Kirmani, Nuzhat Noureen Rana, Osama Atawneh, George Koutsis, Marianthi Breza, Salvatore Mangano, Carmela Scuderi, Eugenia Borgione, Giovanna Morello, Tanya Stojkovic, Massimi Zollo, Gali Heimer, Yves A. Dauvilliers, Pasquale Striano, Issam Al-Khawaja, Fuad Al-Mutairi, Hamed Sherifa.

## Abbreviations

GAD =: glutamate decarboxylase
