# Supplementary material for: Early-infantile onset epilepsy and developmental delay caused by bi-allelic GAD1 variants
Source: Brain. 2020 Jul 23;143(8):2388–97. doi: 10.1093/brain/awaa178 (PMC7447512; doi:10.1093/brain/awaa178)
Supplement: awaa178_Supplementary_Data [file awaa178_supplementary_data.zip › awaa178-suppl_data/brain-2019-02230-File004.pdf]

SUPPLEMENTARY MATERIAL

Supplementary methods

This study was approved by local institutional IRB/ethical review boards, and written informed consent was obtained prior to genetic testing from the family involved.

Clinical details were obtained through medical file review and clinical examination.

Genomic DNA was extracted from peripheral blood samples according to standard procedures of phenol chloroform extraction. WES on the proband was performed as described elsewhere (Mencacci et al. 2016) in Macrogen, Korea. Briefly, target enrichment was performed with 2 μg genomic DNA using the SureSelectXT Human All Exon Kit version 6 (Agilent Technologies, Santa Clara, CA, USA) to generate barcoded whole-exome sequencing libraries. Libraries were sequenced on the HiSeqX platform (Illumina, San Diego, CA, USA) with 50x coverage. Quality assessment of the sequence reads was performed by generating QC statistics with FastQC (http://www.bioinformatics.bbsrc.ac.uk/projects/fastqc).

Our bioinformatics filtering strategy included screening for only exonic and donor/acceptor splicing variants. In accordance with the pedigree and phenotype, priority was given to rare variants (<0.01% in public databases, including 1,000 Genomes project, NHLBI Exome Variant Server, Complete Genomics 69, and Exome Aggregation Consortium [ExAC v0.2]) that were fitting a recessive (homozygous or compound heterozygous) or a de novo model and/or variants in genes previously linked to developmental delay, intellectual disability and other neurological disorders.

Using genomic DNA from the proband and parents, the exonic regions and flanking splice junctions of the genome were captured using the IDT xGen Exome Research Panel v1.0. Massively parallel (NextGen) sequencing was done on an Illumina system with 100bp or greater paired-end reads. Reads were aligned to human genome build GRCh37/UCSC hg19, and analyzed for sequence variants using a custom-developed analysis tool. Additional sequencing technology and variant interpretation protocol has been previously described (Retterer et al. 2016). The general assertion criteria for variant classification are publicly available on the GeneDx ClinVar submission page (http://www.ncbi.nlm.nih.gov/clinvar/submitters/26957/).

Web resources

The following databases and prediction scores were used in this study:

UCSC Human Genome Database: http://www.genome.UCSC.edu

Greater Middle East (GME) Variome web: http://igm.ucsd.edu/gme/index.php

Genome Aggregation Database (gnomAD): http://gnomad.broadinstitute.org/

Exome Aggregation Consortium (ExAC): http://exac.broadinstitute.org/

Gene Matcher (GeneMatcher): https://genematcher.org/

MutationTaster: http://mutationtaster.org/

Iranome: www.iranome.ir

Ensembl: https://www.ensembl.org

SIFT: https://sift.bii.a-star.edu.sg/

Polyphen-2: http://genetics.bwh.harvard.edu/pph2/

CADD score: https://cadd.gs.washington.edu/

FOLDX suite: http://foldxsuite.crg.eu/

Missense 3D: http://www.sbg.bio.ic.ac.uk/~missense3d/

CONSURF: http://consurf.tau.ac.il/

Supplementary Data

In patient from family F the heterozygous variant c.1403C>T, p.(Ala468Val) in *COL11A2* (NM_080680.3) was also reported. *COL11A2* pathogenic variants cause autosomal dominant otospondylomegaepiphyseal dysplasia (MIM #184840), a condition frequently associated with cleft palate (Melkoniemi et al. 2000; Sirko-Osadsa et al. 1998). However, although predicted damaging by some *in silico* tools (Mutation Taster, SIFT, PROVEAN), others had a benign prediction (e.g., REVEL and DEOGEN2), and this variant has an allele frequency of 0.00001069 in gnomAD (3 heterozygous individuals) and without segregation this is a variant of unknown significance that is unlikely to be pathogenic.

Supplementary figure

Supplementary Table

Table 1: *in silico* prediction of protein stability, protein structure and amino acid conservation of GAD1

	Location	*FoldX* prediction of protein stability	*Missense3D* analysis w/ wt structure	Amino acid conservation based on 150 orthologues (*Consurf*)
p.F324C	Buried in a hydrophobic patch in the PLP domain	Reduces stability (ΔΔG 4.85 kcal/mol)	No obvious structural damage	This AA position is almost invariably Phe
p.T347M	Buried in PLP domain, part of a loop lining the PLP site	No significant change in stability	Breaks some H-bonds	This AA position is predominantly Thr (98%); Met observed in 1 odd orthologue
p.N564S	In C-terminal domain, located at dimer interface, forming H-bond with opposite chain	Reduces stability (ΔΔG 1.47 kcal/mol)	No obvious structural damage	Asn is most commonly observed AA in this position (80% of orthologues); Ser observed in 4% of orthologues

Supplementary References

Mencacci, Niccolò E.; Kamsteeg, Erik-Jan; Nakashima, Kosuke; R'Bibo, Lea; Lynch, David S.; Balint, Bettina et al. (2016): De Novo Mutations in PDE10A Cause Childhood-Onset Chorea with Bilateral Striatal Lesions. In: *American journal of human genetics* 98 (4), S. 763–771. DOI: 10.1016/j.ajhg.2016.02.015.

Retterer, Kyle; Juusola, Jane; Cho, Megan T.; Vitazka, Patrik; Millan, Francisca; Gibellini, Federica et al. (2016): Clinical application of whole-exome sequencing across clinical indications. In: *Genetics in medicine : official journal of the American College of Medical Genetics* 18 (7), S. 696–704. DOI: 10.1038/gim.2015.148.

Literaturverzeichnis

Melkoniemi, M.; Brunner, H. G.; Manouvrier, S.; Hennekam, R.; Superti-Furga, A.; Kääriäinen, H. et al. (2000): Autosomal recessive disorder otospondylomegaepiphyseal dysplasia is associated with loss-of-function mutations in the COL11A2 gene. In: *American journal of human genetics* 66 (2), S. 368–377. DOI: 10.1086/302750.

Sirko-Osadsa, D. A.; Murray, M. A.; Scott, J. A.; Lavery, M. A.; Warman, M. L.; Robin, N. H. (1998): Stickler syndrome without eye involvement is caused by mutations in COL11A2, the gene encoding the alpha2(XI) chain of type XI collagen. In: *The Journal of pediatrics* 132 (2), S. 368–371. DOI: 10.1016/s0022-3476(98)70466-4.
