# Supplementary material for: Early-infantile onset epilepsy and developmental delay caused by bi-allelic GAD1 variants
Source: Brain. 2020 Jul 23;143(8):2388–97. doi: 10.1093/brain/awaa178 (PMC7447512; doi:10.1093/brain/awaa178)
Supplement: awaa178_Supplementary_Data [file awaa178_supplementary_data.zip › awaa178-suppl_data/brain-2019-02230-File008.pdf]

Consortia and networks involved in this study:

The Synaptopathies and Paroxysmal Syndromes (SYNaPS) Study Group (http://neurogenetics.co.uk/synaptopathies-synaps/)

*Study Group Members*:

Prof Stanislav Groppa, Department of Neurology and Neurosurgery, Institute of Emergency Medicine, Chisinau, Republic of Moldova; sgroppa@gmail.com

Dr. Blagovesta Marinova Karashova, Department of Paediatrics, Medical University of Sofia, Sofia 1431, Bulgaria; blagovestakarashova@gmail.com

Dr. Wolfgang Nachbauer, Department of Neurology, Medical University Innsbruck, Anichstrasse 35, Innsbruck 6020, Austria; Wolfgang.Nachbauer@i-med.ac.at

Prof. Sylvia Boesch, Department of Neurology, Medical University Innsbruck, Anichstrasse 35, Innsbruck 6020, Austria; sylvia.boesch@i-med.ac.at

Dr. Larissa Arning, Department of Human Genetics, Ruhr-University Bochum, Bochum 44801, Germany; Larissa.Arning@ruhr-uni-bochum.de

Prof. Dagmar Timmann, Braun Neurologische Universitätsklinik Universität Essen, Hufelandstr 55, Essen D-45122, Germany; Dagmar.Timmann-Braun@uni-duisburg-essen.de

Prof. Bru Cormand, Department of Genetics, Universitat de Barcelona, Barcelona 08007, Spain; bcormand@ub.edu

Dr. Belen Pérez-Dueñas, Hospital Sant Joan de Deu, Esplugues de Llobregat 08950, Barcelona, Spain; bperez@sjdhospitalbarcelona.org

Dr Gabriella Di Rosa, MD, PhD, Department of Pediatrics, University of Messina, Messina 98123, Italy; gdirosa@unime.it

Prof. Jatinder S. Goraya, MD, FRCP, Division of Paediatric Neurology, Dayanand Medical College & Hospital, Ludhiana, Punjab 141001, India; gorayajs@gmail.com

Prof. Tipu Sultan, Division of Paediatric Neurology, Children's Hospital of Lahore, Lahore 381-D/2, Pakistan; tipusultanmalik@hotmail.com

Prof Jun Mine, Department of Paediatrics, Shimane University, Faculty of Medicine, Izumo, 693-8501, Japan; jmine@med.shimane-u.ac.jp

Prof. Daniela Avdjieva, Department of Paediatrics, Medical University of Sofia, Sofia 1431, Bulgaria; davdjieva@yahoo.com

Dr. Hadil Kathom, Department of Pediatrics, Medical University of Sofia, Sofia 1431, Bulgaria; hadilmk@gmail.com

Prof.Dr Radka Tincheva, Head of Department of Clinical Genetics, University Pediatric Hospital, Sofia 1431, Bulgaria; radka.tincheva@gmail.com

Prof. Selina Banu, Neurosciences Unit, Institute of Child Health and Shishu Shastho Foundation Hospital, Mirpur, Dhaka 1216, Bangladesh; selinabanu17@gmail.com

Prof. Mercedes Pineda-Marfa, Servei de Neurologia Pediàtrica, l'Hospital Universitari Vall d'Hebron, Barcelona 08035, Spain; pineda@hsjdbcn.org

Prof. Pierangelo Veggiotti, Unit of Infantile Neuropsychiatry Fondazione, Istituto Neurologico "C. Mondino" IRCCS, Via Mondino 2, Pavia 27100, Italy; pierangelo.veggiotti@unipv.it

Prof. Michel D. Ferrari, Leiden University Medical Center, Albinusdreef 2, Leiden 2333, Netherlands; M.D.Ferrari@lumc.nl

Prof. Alberto Verrotti , University of L’Aquila, L’Aquila, Italy; verrottidipianella@univaq.it

Prof Giangluigi Marseglia, Department of Pediatrics, University of Pavia, IRCCS Policlinico "San Matteo", Pavia 27100, Italy
Email: gl.marseglia@smatteo.pv.it

Dr. Salvatore Savasta, Division of Pediatric Neurology, Department of Pediatrics, University of Pavia, IRCCS Policlinico "San Matteo", Pavia 27100, Italy; S.Savasta@smatteo.pv.it

Dr. Mayte García-Silva, Hospital Universitario 12 de Octubre, Madrid 28041, Spain, mgarciasilva@salud.madrid.org

Dr. Alfons Macaya Ruiz, University Hospital Vall d'Hebron, Barcelona 08035, Spain, amacaya@vhebron.net

Prof. Barbara Garavaglia, IRCCS Foundation, Neurological Institute “Carlo Besta”, Molecular Neurogenetics, 20126 Milan, Italy; segr.neurogenetica@istituto-besta.it

Dr. Eugenia Borgione, Laboratorio di Neuropatologia Clinica, U.O.S. Malattie, Neuromuscolari Associazione OASI Maria SS. ONLUS – IRCCS, Via Conte Ruggero 73, 94018 Troina, Italy; eborgione@oasi.en.it

Dr. Simona Portaro, IRCCS Centro Neurolesi "Bonino Pulejo", SS113, c.da Casazza, 98124 Messina, Italy; simonaportaro@hotmail.it

Dr. Benigno Monteagudo Sanchez, Hospital Arquitecto Marcide, Avenida de la Residencia S/N, Ferrol (A Coruña), 15401 Spain
Email: benims@hotmail.com

Dr. Richard Boles, Courtagen Life Sciences, 12 Gill Street Suite 3700, Woburn, MA 01801 USA; Richard.Boles@courtagen.com

Prof. Savvas Papacostas, Neurology Clinic B, The Cyprus Institute of Neurology and Genetics, 6 International Airport Road, 1683 Nicosia, Cyprus; savvas@cing.ac.cy

Dr. Michail Vikelis, Iatreio Kefalalgias Glyfadas, 8 Lazaraki str, 3rd floor, 16675, Athens, Greece; mvikelis@headaches.gr

Prof Eleni Zamba Papanicolaou, The Cyprus Institute of Neurology & Genetics, Nicosia, Cyprus; ezamba@cing.ac.cy

Dr Efthymios Dardiotis, UNIVERSITY HOSPITAL OF LARISSA, NEUROLOGY Department, Greece; edar@med.uth.gr

Prof Shazia Maqbool, Department of Developmental and Behavioral Pediatrics, CH&ICH, Lahore, Pakistan; drshazimaq@yahoo.com

Prof Shahnaz Ibrahim, Department of Pediatrics and child health, Aga Khan University, Karachi, Pakistan; shahnaz.ibrahim@aku.edu

Prof Salman Kirmani, Department of Paediatrics & Child Health, The Aga Khan University, Karachi , Pakistan; salman.kirmani@aku.edu

Dr. Nuzhat Noureen Rana, Department of Paediatric Neurology, Children Hospital Complex and ICH, Multan, Pakistan; drnuzhatrana@gmail.com

Dr. Osama Atawneh, Hilal Pediatric Hospital Hebron, Hebron West Bank, Palestine; osamaat@gmail.com

Prof George Koutsis, Dr Marianthi Breza, Neurogenetics Unit, Neurology Department, Eginition Hospital, National and Kapodistrian University, Athens, Greece; marianthibr@med.uoa.gr

Prof Salvatore Mangano, Unità di Neuropsichiatria Infantile, AOUP "P.Giaccone" Palermo, Italy; salvatore.mangano@unipa.it

Dr Carmela Scuderi, Associazione Oasi Maria SS, 94018 Troina, Italy; cscuderi@oasi.en.it

Dr Eugenia Borgione, Associazione Oasi Maria SS, 94018 Troina, Italy; eborgione@oasi.en.it

Dr Giovanna Morello, Institute of Neurological Sciences, National Research Council, Mangone, Italy; g.morello@isn.cnr.it

Dr Tanya Stojkovic, Institute of Myology, Hôpital La Pitié Salpêtrière, Paris, France; stojkovic.tanya@aphp.fr

Prof Massimi Zollo, CEINGE, Biotecnologie Avanzate S.c.a.rl., Naples, Italy; massimo.zollo@unina.it

Dr Gali Heimer, University Hospital of Tel Aviv, Tel Aviv, Israel; galih.md@gmail.com

Prof Yves A. Dauvilliers, University Hospital Montpellier, Montpellier, France; ydauvilliers@yahoo.fr

Prof Pasquale Striano, Institute “Giannina Gaslini”, Genova, Italy; strianop@gmail.com

Dr Issam Al-Khawaja, Albashir University Hospital, Amman, Jordan; isamkhawaja61@gmail.com

Dr Fuad Al-Mutairi, King Saud University, Riyadh, Saudi Arabia; almutairifu@NGHA.MED.SA

Prof Hamed Sherifa, Assiut University Hospital, Assiut, Egypt; hamed_sherifa@yahoo.com
